# Oxidative Phosphorylation Impairment by DDT and DDE

**DOI:** 10.3389/fendo.2019.00122

**Published:** 2019-03-12

**Authors:** Sarah E. Elmore, Michele A. La Merrill

**Affiliations:** Department of Environmental Toxicology, University of California, Davis, Davis, CA, United States

**Keywords:** mitotoxicity, electron transport chain, insulin resistance, obesity, DDT, DDE, pesticides

## Abstract

There is increasing evidence supporting the characterization of the pesticide DDT and its metabolite, DDE, as obesogens and metabolic disruptors. Elucidating the mechanism is critical to understanding whether the association of DDT and DDE with obesity and diabetes is in fact causal. One area of research investigating the etiology of metabolic diseases is mitochondrial toxicity. Several studies have found associations between mitochondrial defects and insulin resistance, cellular respiration, substrate utilization, and energy expenditure. Although the mitotoxicity of DDT and DDE was established 20–40 years ago, it was not viewed in the light of the diseases faced today; therefore, it is prudent to reexamine the mitotoxicity literature for mechanistic support of DDT and DDE as causal contributors to obesity and diabetes, as well as associated diseases, such as cancer and *Alzheimer's* disease. This review aims to focus on studies investigating the effect of DDT or DDE on mammalian mitochondrial oxidative phosphorylation. We illustrate that both DDT and DDE impair the electron transport chain (ETC) and oxidative phosphorylation. We conclude that there is reasonable data to suggest that DDT and DDE target specific complexes and processes within the mitochondria, and that these insults could in turn contribute to the role of DDT and DDE in mitochondria-associated diseases.

## Introduction

The discovery of dichlorodiphenyltrichloroethane (DDT) as an efficient insecticide won Paul Muller the Nobel Prize in the 1940s. Initially used to control vector-borne diseases, such as malaria, its broad use as a pesticide quickly grew. However, DDT was banned in 1972 in the United States due to adverse environmental effects. Although banned by many countries following the 2001 Stockholm Convention, DDT is still recommended for indoor residual spraying to control malaria vectors by the World Health Organization (1) and as such, continues to be manufactured and used. Current US FDA guidelines limit DDT and DDE levels to 0.05–5 ppm depending on the commodity (2).

DDT and its metabolite dichlorodiphenyldicholorethylene (DDE) are both persistent organic pollutants (POPs) due to their physiochemical properties, allowing for biomagnification and their storage in the lipid-rich adipose tissue of mammals (3, 4). The environmental persistence of DDT and DDE, combined with the fact that DDT is still manufactured and used in parts of the world today, make DDT and DDE relevant public health concerns.

In a recent integrated systematic review and meta-analysis, *p,p*′-DDT and *p,p*′-DDE were classified as “presumed” to be obesogenic for humans, based on prospective epidemiological observations integrated with experimental evidence of increased rodent adiposity and impaired energy expenditure (5). Numerous studies have suggested that exposure to DDT and/or DDE are additionally associated with several diseases linked to obesity, namely type 2 diabetes (T2D), Alzheimer's disease (AD), and cancer (6–11). However, the mechanism of impairment by DDT or DDE which leads to these diseases remains unresolved. One supporting mechanistic hypothesis is that DDT and DDE are mitotoxicants. Indeed, the role of POPs, including DDT and DDE, in mitochondrial dysfunction and metabolic diseases, such as obesity and T2D has been broadly reviewed (12–14). Furthermore, subtle mitochondrial malfunctions appear to be involved in the pathogenesis of insulin resistance, T2D, AD, and cancer. However, specific mitochondrial targets of DDT or DDE have not been examined across the existing literature to our knowledge.

The predominant function of mitochondria is the generation of ATP by oxidative phosphorylation (OxPhos), but also includes the generation and detoxification of reactive oxygen species, apoptosis, regulation of calcium, metabolism, self-transportation, and thermogenesis (15, 16). Thorough reviews of the methods available to assess mitochondrial dysfunction are available (15, 17).

**Figure 1:**
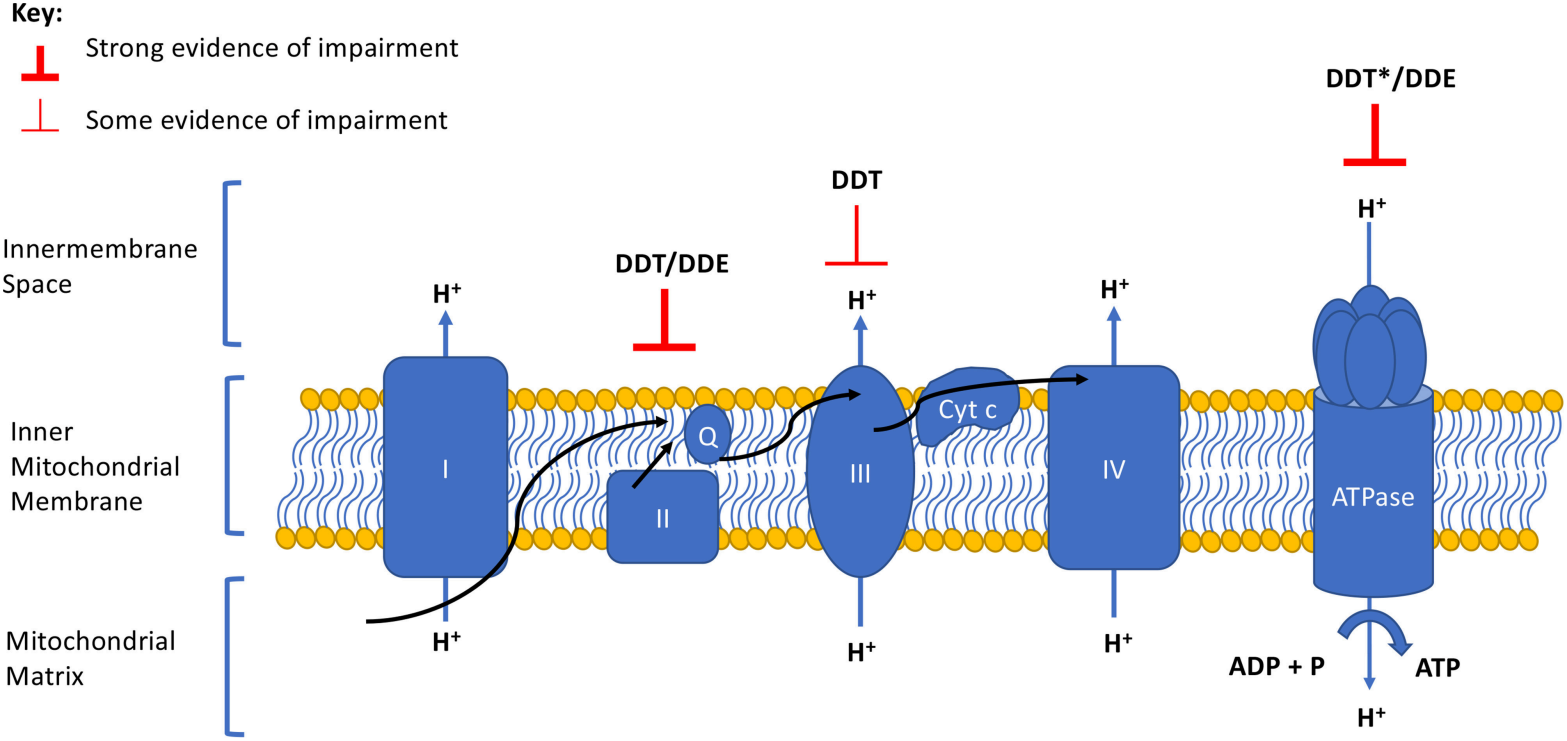
Summary of DDT and DDE effects on the electron transport chain and oxidative phosphorylation complexes. Degree of evidence is represented by weight of line and corresponds to data presented in Table 1. Arrows indicate the direction of the electron flow. Q, ubiquinone; Cyt *c*, cytochrome *c*. ^*^Evidence of a strong effect but opposing directionality exists.

Impaired cellular respiration (18–21) and mitochondrial membrane potential (18, 20) have been observed in mammalian mitochondria after exposure to DDT and DDE. DDE also decreased membrane potential, ATP levels, and oxygen consumption rates in human HepG2 cells (22). In this review, we summarize the mechanistic evidence supporting these mitotoxicities by focusing on studies investigating the effect of DDT or DDE on OxPhos, specifically the complexes of the ETC and the efficiency of coupling ATP synthesis to the ETC. We illustrate that both DDT and DDE impair specific complexes of the ETC that contribute to an overall reduction in OxPhos (Table 1 and Figure 1). Although other chemicals may interact with DDT or DDE in targeting OxPhos, exploration of mixture effects is out of the scope of this mini-review.

**Table 1:** Summary of the effect of DDT and DDE on oxidative phosphorylation.

**Purity**	**Model**	**Dose range**	**LOEL**	**Complex I**	**Complex II**	**Complex III**	**Complex IV**	**ATP synthase**	**Uncoupling**	**Morphological or structural changes**	**References**
**DDT**											
Not listed	Heavy beef heart mitochondria	~2.5 umol/mg^a^	~2.5 umol/mg	0–15% depression of oxidase activity by 1 umol in heavy heart beef mitochondria.	Reduction in succinate dehydrogenase enzymatic activity by 10–20% with 1 umol DDT in beef mitochondria.	—	—	—	—	—	(23)
>99% by gas-liquid chromatography	Rat liver mitochondria	0, 50, 100, 150, 200 uM	10 uM	X	16% inhibition of succinate dehydrogenase with 50 uM.	39% inhibition of succinate-cytochrome c reductase with 50 uM; 50 nmol/mg interferes with electron transfer between cytochromes b and c.	X	DDT inhibits ATPase by 38% with 200 uM in isolated mitochondria as measured by Ca^2+^ uptake driving by ATP hydrolysis. Inhibition starting around 10 uM.	Uncoupling observed with >200 uM in the presence of TMPD.	—	(24)
Not listed	Rat liver mitochondria	0, 10, 20, 30, 40, 50 nmol DDT/mg	20 nmol/mg	X	X	Inhibition of succinate cytochrome c reductase in isolated rat mitochondria after 20 nmol/mg; secondary effect on the ubiquinol-cytochrome c subunit.	X	Decrease in ATP synthesis at 3 nmol/mg. Increase in ATPase activity with ≥9.4 nmol/mg as measured by monitoring pH associated with ATP hydrolysis; inhibitory action at 19 nmol/mg on ATPase as measured by membrane potential.	X	Swelling observed above 50 nmol/mg.	(18)
>99% as tested by gc and hplc	Rat submitochondria	30 ug/ml	31 ug/ml	—	—	—	—	DDT inhibited ATPase activity by 29.4%.	—	—	(19)
>99% as tested by gc and hplc	Rat liver mitochondria	0, 4, 8, 20 ug/ml	4 ug/ml	—	—	—	—	Increase of latent ATPase activity at all doses.	Uncoupling observed with stimulation of State 4 respiration around 10 ug/ml.	Mitochondrial Swelling observed with 10 and 20 ug/ml.	(19)
Analytical grade	Rat liver mitochondria	0, 50, 200, 600 mg/kg	600 mg/kg	—	—	—	—	Increase in ATPase activity in rat liver and brain mitochondria by 600 mg/kg.	—	—	(21)
**References in agreement**				2/3	2/3	2/2	2/2	Conflicting agreement	2/3	2/2	
**Evidence determination**				Equivocal evidence–additional species needed	Equivocal evidence–additional species needed	Strong evidence that implicates transfer from Complex II	No evidence of effect	Strong evidence in a sensitive system	Equivocal evidence; unrealistic conditions	Strong evidence; unrealistic conditions	
**DDE**											
Not listed	Heavy beef heart mitochondria	~2.5 umol/mg	~2.5 umol/mg	5–20% depression of oxidase activity by 1 umol in heavy heart beef mitochondria.	Reduction in succinoxidase enzymatic activity by 5–15% with 1 umol DDE in beef mitochondria.	—	—	—	—	—	(23)
>99% as tested by gc and hplc	Rat Submitochondria	30 ug/ml	30 ug/ml	—	—	—	—	DDE inhibited ATPase activity by 32.4%.	—	—	(19)
>99% as tested by gc and hplc	Rat liver mitochondria	0, 4, 8, 20 ug/ml	4 ug/ml	—	—	—	—	Increase of latent ATPase activity at all doses.	Uncoupling observed with stimulation of State 4 respiration around 10 ug/ml.	Mitochondrial Swelling observed with 10 and 20 ug/ml.	(19)
Chromatographic grade	Rat liver mitochondria	0, 20, 30 40, 50, 80, 100 nmol/mg protein	50 nmol/mg	X	Succinate dehydrogenase and succinate cytochrome *c* reductase were partially inhibited with 50 nmol DDE/mg.	X	X	ATPase activity is inhibited at does below 50 nmol/mg when succinate is present. DDE stimulated ATPase in the presence of an uncoupler only at high concentrations (80 nmol/DDE/mg protein).	>80 nmol/mg increased permeability to protons, uncoupling oxidation from phosphorylation.	Mitochondrial Swelling observed with 80 nmol DDE/mg.	(20)
**References in agreement**				0/2	2/2	1/1	1/1	Conflicting agreement	2/2	2/2	
**Evidence determination**				Equivocal evidence–additional species needed	Strong evidence	No evidence of effect	No evidence of effect	Strong evidence in a sensitive system	Strong evidence; unrealistic conditions	Strong evidence; unrealistic conditions	

a *Approximate values based on a range of 0.36–0.42 mg of protein for 1 umol of DDT; X indicate no significant effect reported; — indicate complex not studied*.

## Complex I

Complex I (Figure 1) is also known as the NADH dehydrogenase complex or NADH:ubiquinone oxidoreductase. This enzyme complex is responsible for accepting electrons from NADPH and ultimately passing them to the next complex through ubiquinone as protons are pumped across the inner mitochondrial membrane. Systems that are impaired at this complex would have a depression in mitochondrial potential and cellular respiration resulting in one less proton (ATP) produced by the energy transfer of the electron.

Pardini et al. (23) reported a depression of 0–15% of baseline NADH dehydrogenase activity by 2.5 umol DDT/mg of mitochondria from heavy beef heart. However, others did not observe significant defects at Complex I in isolated rat mitochondria at doses above or below that dose (18, 24). Based on the studies reviewed here, there is equivocal evidence implicating a DDT impairment at Complex I. Additional species and conditions are necessary to make a further determination.

Pardini et al. (23) also reported a 5–20% depression of baseline NADH dehydrogenase activity by 2.5 umol/mg of DDE in heavy beef heart mitochondria. Yet Ferreira et al. (20) did not report any changes at Complex I in rat mitochondria exposed to the same range of DDE doses. Without additional evidence it is difficult to reach a conclusion regarding the possible effects of DDE on Complex I; additional studies are necessary to determine the directionality of a DDE effect.

## Complex II

Complex II is contains the enzyme succinate dehydrogenase, also known succinate-CoQ reductase. At this Complex, additional electrons are delivered from the substrate succinate to a quinone pool via flavin adenine dinucleotide (FAD). No protons are transported across the intermembrane space, hence a defect at Complex II would result in a depression of cellular respiration but no change in proton motive force.

Moreno and Madeira (18) reported that Complex II is insensitive to DDT. Conversely, Pardini et al. (23) observed that succinate dehydrogenase enzymatic activity was reduced by 10–20% after heavy beef mitochondria were treated with DDT and Nishihara and Utsumi (24) observed a 16% inhibition of succinate dehydrogenase in rat mitochondria after treatment with 50 uM of DDT. Given that Complex II does not contribute to changes in mitochondrial membrane potential, the weak defects by DDT reported here are unlikely to be responsible for the decreased mitochondrial membrane potential observed in mammalian mitochondria exposed to DDT and DDE (18, 20), but may cause OxPhos impairment through reduced substrate transfer from Complex I to Complex II (Figure 1).

Pardini et al. (23) also observed enzymatic depressions at Complex II by DDE in heavy beef mitochondria. More compelling yet, while Ferreira et al. (20) reported a depression in respiration and membrane potential in the presence of the Complex II substrate succinate at 10 nmol of DDE in isolated rat mitochondria, this outcome was not observed when Complex III substrates were used to bypass Complex II defects, e.g., ascorbate and the cytochrome *c* electron donator *N*,*N*,*N*′,*N*′-Tetramethyl-pphenylenediamine dihydrochloride (TMPD). The full restoration of respiration and membrane potential by ascorbate and TMPD suggests that inhibition of Complex II and inhibition of succinate translocation is the source of DDE depression of mitochondrial respiration. This evidence supports the inference that the inhibitory effects of DDE on Complex II contribute to a limited capacity for ATP production through substrate transfer rather than the proton gradient, to cause an energy imbalance (Figure 1).

## Complex III

Complex III, also referred to as cytochrome *b*-*c*_1_, contains at least 11 different polypeptide chains and functions as a dimer. This complex accepts electrons from the substrate ubiquinone and passes them cytochrome *c*, which carries its electron to Complex IV (one electron per cytochrome *c*); ubiquinol—cytochrome-c reductase catalyzes the chemical reaction. At this Complex, protons are transferred to the inner membrane space, contributing to membrane potential.

Nishihara and Utsumi (24) reported that DDT interfered with electron transfer via a 39% inhibition of succinate-cytochrome c reductase activity after treatment of rat-liver mitochondria with 50 uM DDT. The authors concluded that this defect originated at the electron transfer between cytochrome *b* and *c*. This effect was only observed when succinate was supplied as the substrate, suggesting that the overall electron transfer defect resulted from a combination of defects at Complex II and III of the ETC. In support of DDT interference with activity between cytochromes *b* and *c*, Moreno and Madeira (18) observed direct inhibition of the ubiquinol-cytochrome *c* subunit of isolated rat mitochondria.

Cytochrome *c* oxidase activity was not impaired when TMPD (serving as a Complex III substrate that bypasses Complex II through its cytochrome c electron donation) was used as the substrate after DDE exposure (20). This observation supports the notion that Complex III machinery and function was intact and working properly after DDE exposure.

## Complex IV

Complex IV, also known as cytochrome *c* oxidase, is the segment where four electrons are removed from four molecules of cytochrome *c* and transferred to oxygen to produce two water molecules. Simultaneously, protons are moved from the mitochondrial matrix to the inner membrane thus contributing to the mitochondrial proton gradient.

Both Nishihara and Utsumi (24) and Moreno and Madeira (18) reported that DDT did not affect the cytochrome c oxidase segment of Complex IV.

Ferreira et al. (20) found that Complex IV of the ETC was not affected by DDE.

## ATP Synthase (Complex V)

ATP Synthase (ATPase), often referred to as Complex V, is the final segment of the ETC. It transports a proton into the inner mitochondrial space as energy to increase the proton gradient which fuels the phosphorylation of ADP to ATP.

DDT appears to act on ATPase in every study that has examined it, but the direction of effect is inconsistent (Table 1). On one hand, several authors reported that DDT stimulated ATPase activity in both rat liver and brain mitochondria after DDT treatment (18, 21). Similarly, Ohyama et al. (19) reported that DDT stimulated ATPase residing in intact mitochondria yet they also observed an inhibitory effect of DDT on ATPase from sonicated submitochondria particles. These results suggest DDT can inhibit ATPase when ATPase is uncoupled from the ETC. However, other studies of intact mitochondria suggest DDT can inhibit ATPase even when coupled to ETC. For example, Nishihara and Utsumi (24) evaluated isolated rat mitochondria and found weak inhibition of ATPase by DDT starting at 10 uM, with maximum inhibition (38% of control) observed with 200 uM DDT. Further, Moreno and Madeira (18) reported inhibited ATPase activity and decreased ATP synthesis in isolated rat mitochondria exposed to low dose DDT.

These differing effects of DDT on ATPase across different methods implemented by Moreno and Madeira may reflect confounding effects by unreported experimental parameters, such as temperature. For example, the motor protein that couples ATP hydrolysis to mechanical rotation was recently characterized by Wantanabe and Noji (25), who found that the rotation of ATPase is highly temperature sensitive. However, despite experimental differences, the consistent perturbation of ATPase activity by DDT among the body of evidence resulting from examination of the effects of DDT on ATPase suggests that ATPase is a target of DDT toxicity and may result in some sort of energy dissipation through Complex V.

Similar to DDT, DDE appears to act on ATPase in most studies reviewed here, but the direction of the DDE effect is inconsistent (Table 1). For example, Ohyama et al. (19) reported a stimulation of “latent” ATPase activity by DDE (4–20 ug/ml) in isolated rat mitochondria and a DDE (30 ug/ml) inhibition of ATPase when uncoupled from the ETC in submitochondrial factions. Conversely, Ferreira et al. (20) did not observe any ATPase defects when isolated mitochondria or submitochondrial particles were exposed to 20 or 50 nmol DDE/mg. Once again, it is reasonable to suggest that ATPase may be a target of DDE toxicity and result in some sort of energy dissipation through the enzyme complex.

## ETC Uncoupling

In Complex V, ATPase is responsible for “coupling” the proton gradient of the ETC to ATP synthesis. This process can be uncoupled when uncoupling protein leaks protons back into the inner mitochondrial matrix generating heat rather than producing ATP. Conversely, non-canonical uncoupling can occur in the absence of electron flow and ATPase inhibition, when for other reasons, ATP synthesis cannot take place. These reasons include exposure to uncoupling agents, such as FCCP or CCCP, or to physical force, such as osmotic shock, that dissipates the pH or membrane potential of the mitochondria (26).

The literature suggests that DDT does not uncouple OxPhos. Moreno and Madeira (18) reported that only large concentrations of DDT caused extensive proton leak. This is consistent with the uncoupled OxPhos by high doses of DDT observed by Ohyama et al. (19) and by Nishihara and Utsumi (24). Given the lipophilicity of DDT, these observations of uncoupled OxPhos following high dose exposure to DDT likely reflect nothing more than non-specific destruction of the mitochondrial membrane.

Similar to DDT experimental outcomes, Ferreira et al. (20) observed partial uncoupling of OxPhos in isolated rat mitochondria at high doses of DDE (>80 nmol/mg protein). Ohyama et al. (19) came to a similar conclusion after reporting uncoupling activity that resulted in stimulation of State 4 respiration. Ferreira and Ohyama suggest that this effect is likely due to disruption of the mitochondrial inner membrane by the high, non-biologically relevant, doses of DDE used.

## Summary of Results and Discussion of the Research Gaps

The *in vitro* studies, primarily in rodent mitochondria, discussed in this review clearly demonstrate the toxic effects of DDT and DDE on Complex II and Complex V of the ETC. Toxicity to Complex II appears to result from substrate disruption. Indeed, given there is no proton transport by Complex II, if DDT and DDE target Complex II, the resulting Complex II perturbation does not explain the reported effects on reduced membrane potential. Instead, we suspect that disruption to ATPase activity by DDT and DDE may contribute to defects associated with mitochondrial respiration and membrane potential. Although inconsistencies in the effects of DDT and DDE on ATPase remain to be resolved, it is important to note that ATPases vary in their sensitivity to DDT depending on temperature (19); this could contribute to different results across systems tested.

Early work presented by Byczkowski (21) suggest mitochondrial uncoupling was the mode of action for DDT mitotoxicity, however it appears this was only the case when DDT or DDE levels exceeded 50 nmol/mg; doses at or above this level often coincided with mitochondrial swelling, an indicator of mitochondrial dysfunction resulting from mitochondrial permeability (27).

Through this review, several mechanistic gaps of DDT and DDE mitotoxicity became apparent. First, given most studies investigated mitochondria from rats and their livers, there is a need for the demonstration of consistency of DDT and DDE mitotoxicity across multiple species and tissues. Given mitochondrial functions vary by cell type and the emerging relationships between DDT, mitochondrial-dense brown adipose tissue, and obesity [e.g., (28)], this is a tissue in need of characterization. Additionally, given the evidence supporting a role of substrate perturbation in Complex II toxicity caused by DDT and DDE, whole cell and/or ETC substrate studies should be conducted. Lastly, the direction of DDT and DDE effects on ATPase function should be resolved at doses more relevant to the human condition. In the meta-analysis of prospective human studies associating DDTs with obesity, Cano-Sancho et al. (5) found internal concentrations of DDT and DDE to be between 0.001 and 10 ng DDTs/mL. Based on lipid weight conversion as described by Cano-Sancho et al. (5), these obesogenic levels correspond to ~0.001–30 nM of DDT or DDE for *in vitro* dosing. We further suggest that temperature and perhaps pressure be systematically controlled in this endeavor to resolve discrepancies in the DDT and DDE effect at ATPase.

## Implications for Disease Etiology

An increasing number of studies suggest a strong role for DDT and/or DDE in the etiology of human disease including obesity, T2D, AD, and cancer. Based on this review, mitotoxic effects targeting OxPhos appear to be a likely consequence of DDT or DDE exposure which could contribute to the pathogenesis of such diseases.

Obesity is the result of disturbances in energy balance. Rates of obesity are rising in humans and other animals, including primates and rodents serving as experimental controls, feral rodents, and domestic dogs and cats (29) suggesting an etiology beyond overeating and/or inactivity. One source of disturbance in energy metabolism is mitochondrial dysfunction, given the organelle's central role in ATP production and energy expenditure including consequences on lipid and glucose metabolism (30–32). Moreover, the term obesogen has been coined for toxicants that cause such disturbances. Based on meta-analysis of human prospective studies and bioassays, DDT and DDE have been presumed to be obseogens (5). Developmental DDT exposure increased rodent obesity in subsequent generations, where it impaired thermogenesis and decreased energy expenditure while reducing RNA coding for mitochondrial control of thermogenesis and energy expenditure in mice (28, 33).

Similar to obesity trends, the prevalence of T2D has risen dramatically in countries of all incomes (34). T2D is characterized by defects in both insulin action and insulin secretion with emerging evidence that mitochondria dysfunction causes both (35). For example Petersen et al. (36) used 13^C^ and 31^P^ magnetic resonance spectroscopy to demonstrate that insulin resistance could be accompanied by a reduction in mitochondrial oxidative activity and mitochondrial ATP synthesis (36). This mechanism is consistent with work in rodents that demonstrated impaired insulin secretion and action after exposure to DDT (28, 37), and in humans, DDE is associated with T2D (9). The elevated T2D risk observed could arise from decreased mitochondrial membrane potential, ATP levels, and oxygen consumption rates in insulin responsive hepatocytes after DDE exposure (22).

AD is the sixth leading cause of death in the U.S. (38) with poorly understood causes. Its link to mitochondrial activity has recently been explored in a mouse model for familial AD where an age-dependent decrease in mitochondrial complex-II activity starting at 9 months was observed (39). In a separate study of human hippocampal tissues from non-AD controls and AD cases, genes involved in OxPhos were significantly down regulated in subjects with AD including genes involved in both complexes II and V (40). Indeed mitochondrial dysfunction may cause energy failures in neurons to induce synaptic dysfunction underlying cognitive impairment (40). The dysregulation of Complexes II and V by DDT and DDE in the pathogenesis of AD is consistent with two molecular epidemiology studies which found an association between elevated DDE serum levels and AD (8, 41).

In many regards, cancer is a disease of mitochondrial dysfunction characterized by a metabolic shift to anaerobic conditions including mutations in genes encoding mitochondrial proteins (42). DDT has been listed by the California Environmental Protection Agency as an agent causing cancer (43) and classified as “probably carcinogenic to humans” (Group 2A) by the IARC (10, 11), although little has been reported on the mode of DDT's carcinogenic action. Defects in succinate dehydrogenase (complex II), among other mitochondrial enzymes, are associated with both familial and sporadic forms of cancer (42, 44) which is consistent with the effects of DDT and DDE on Complex II reviewed here. DDT and DDE mitotoxicity could hence contribute to at least two key characteristics of cancer (45): (1) through interruption of mitochondrial OxPhos (KC: “induces oxidative stress”) and (2) affecting cellular nutrient supply by altering ATP synthesis (KC: “alters cell proliferation, cell death, or nutrient supply”).

## Conclusion

In summary, there is strong evidence for OxPhos impairment at Complexes II and V by DDT and DDE which in turn could cause or contribute to the etiology of diseases, such as obesity, T2D, AD, and cancer. Future work should consider the experimental details mentioned in this review when investigating the role of DDT and DDE as Complex II and Complex V mitotoxicants as a potential mechanistic causes of these diseases and ideally, use that knowledge to develop therapeutic treatments.

## Author Contributions

ML conceived of the topic, directed and led the literature search, interpreted data, revised manuscript. SE co-led the literature search, interpreted data, drafted, and revised manuscript.

### Conflict of Interest Statement

The authors declare that the research was conducted in the absence of any commercial or financial relationships that could be construed as a potential conflict of interest.
